# Independent risk factors for simvastatin-related myopathy and relevance to different types of muscle symptom

**DOI:** 10.1093/eurheartj/ehaa574

**Published:** 2020-07-23

**Authors:** Jemma C Hopewell, Alison Offer, Richard Haynes, Louise Bowman, Jing Li, Fang Chen, Richard Bulbulia, Mark Lathrop, Colin Baigent, Martin J Landray, Rory Collins, Jane Armitage, Sarah Parish

**Affiliations:** Clinical Trial Service Unit and Epidemiological Studies Unit, Nuffield Department of Population Health, Big Data Institute, University of Oxford, Old Road Campus, Oxford OX3 7LF, UK; Clinical Trial Service Unit and Epidemiological Studies Unit, Nuffield Department of Population Health, Big Data Institute, University of Oxford, Old Road Campus, Oxford OX3 7LF, UK; Clinical Trial Service Unit and Epidemiological Studies Unit, Nuffield Department of Population Health, Big Data Institute, University of Oxford, Old Road Campus, Oxford OX3 7LF, UK; MRC Population Health Research Unit, Nuffield Department of Population Health, University of Oxford, Richard Doll Building, Old Road Campus, Oxford OX3 7LF, UK; Clinical Trial Service Unit and Epidemiological Studies Unit, Nuffield Department of Population Health, Big Data Institute, University of Oxford, Old Road Campus, Oxford OX3 7LF, UK; National Clinical Research Center of Cardiovascular Diseases, State Key Laboratory of Cardiovascular Disease, Fuwai Hospital, National Center for Cardiovascular Diseases, Chinese Academy of Medical Sciences and Peking Union Medical College, Beijing, China; Clinical Trial Service Unit and Epidemiological Studies Unit, Nuffield Department of Population Health, Big Data Institute, University of Oxford, Old Road Campus, Oxford OX3 7LF, UK; Clinical Trial Service Unit and Epidemiological Studies Unit, Nuffield Department of Population Health, Big Data Institute, University of Oxford, Old Road Campus, Oxford OX3 7LF, UK; McGill University and Génome Québec Innovation Centre, 740 Dr Penfield Ave, Montréal, Québec H3A 0G1, Canada; Clinical Trial Service Unit and Epidemiological Studies Unit, Nuffield Department of Population Health, Big Data Institute, University of Oxford, Old Road Campus, Oxford OX3 7LF, UK; MRC Population Health Research Unit, Nuffield Department of Population Health, University of Oxford, Richard Doll Building, Old Road Campus, Oxford OX3 7LF, UK; Clinical Trial Service Unit and Epidemiological Studies Unit, Nuffield Department of Population Health, Big Data Institute, University of Oxford, Old Road Campus, Oxford OX3 7LF, UK; Clinical Trial Service Unit and Epidemiological Studies Unit, Nuffield Department of Population Health, Big Data Institute, University of Oxford, Old Road Campus, Oxford OX3 7LF, UK; Clinical Trial Service Unit and Epidemiological Studies Unit, Nuffield Department of Population Health, Big Data Institute, University of Oxford, Old Road Campus, Oxford OX3 7LF, UK; MRC Population Health Research Unit, Nuffield Department of Population Health, University of Oxford, Richard Doll Building, Old Road Campus, Oxford OX3 7LF, UK; Clinical Trial Service Unit and Epidemiological Studies Unit, Nuffield Department of Population Health, Big Data Institute, University of Oxford, Old Road Campus, Oxford OX3 7LF, UK; MRC Population Health Research Unit, Nuffield Department of Population Health, University of Oxford, Richard Doll Building, Old Road Campus, Oxford OX3 7LF, UK

**Keywords:** Statins, Myopathy, Muscle symptoms, Risk factors, *SLCO1B1*

## Abstract

**Aims:**

Statins are widely used to prevent cardiovascular events, but little is known about the impact of different risk factors for statin-related myopathy or their relevance to reports of other types of muscle symptom.

**Methods and results:**

An observational analysis was undertaken of 171 clinically adjudicated cases of myopathy (defined as unexplained muscle pain or weakness with creatine kinase >10× upper limit of normal) and, separately, of 15 208 cases of other muscle symptoms among 58 390 individuals with vascular disease treated with simvastatin for a mean of 3.4 years. Cox proportional hazards models were used to identify independent predictors of myopathy. The rate of myopathy was low: 9 per 10 000 person-years of simvastatin therapy. Independent risk factors for myopathy included: simvastatin dose, ethnicity, sex, age, body mass index, medically treated diabetes, concomitant use of niacin-laropiprant, verapamil, beta-blockers, diltiazem and diuretics. In combination, these risk factors predicted more than a 30-fold risk difference between the top and bottom thirds of a myopathy risk score (hazard ratio : 34.35, 95% CI: 12.73–92.69, *P* across thirds = 9·1 × 10^−48^). However, despite the strong association with myopathy, this score was not associated with the other reported muscle symptoms (*P* across thirds = 0.93). Likewise, although *SLCO1B1* genotype was associated with myopathy, it was not associated with other muscle symptoms.

**Conclusions:**

The absolute risk of simvastatin-related myopathy is low, but individuals at higher risk can be identified to help guide patient management. The lack of association of the myopathy risk score with other muscle symptoms reinforces randomized placebo-controlled evidence that statins do not cause the vast majority of reported muscle symptoms.

**Figure:**
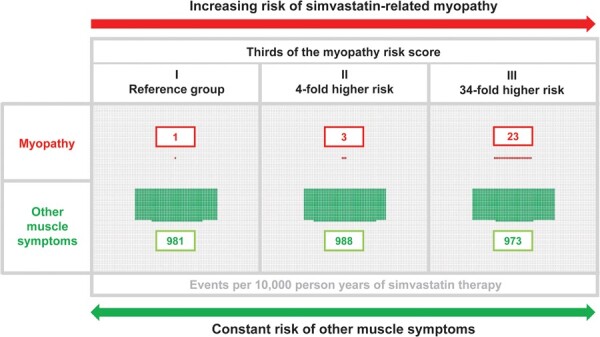

**See page 3343 for the editorial comment on this article (doi: 10.1093/eurheartj/ehaa582)**

## Introduction

Large-scale meta-analyses of randomized controlled trials show that statin therapy reduces the risks of myocardial infarction, coronary revascularization, and ischaemic stroke by about one-fifth for each mmol/L LDL-C reduction, largely irrespective of patient characteristics.^1^ ^,^ ^2^ As a consequence, statins are prescribed to millions of people worldwide, with simvastatin still constituting ∼40% of all statin prescriptions in the UK in 2019 and being the second most commonly prescribed statin in the USA.^3^ ^,^ ^4^

Evidence from randomized controlled trials indicates that statin therapy is safe and well-tolerated.^5^ Statin therapy does rarely cause myopathy, which is a potentially serious side-effect characterized by muscle pain or weakness associated with markedly elevated creatine kinase (CK) levels (e.g. >10× upper limit of normal [ULN]).^5–9^ In contrast, muscle pain or weakness without elevated blood levels of CK is reported as often by patients receiving a statin as by patients receiving matching placebo, suggesting almost all such reports are not caused pharmacologically by the statin (i.e. they are a ‘nocebo’ effect).^5^ ^,^ ^7^ The incidence of myopathy is typically ∼1 per 10 000 person-years with standard statin regimens (such as simvastatin 40 mg daily), but factors that increase blood statin levels—such as higher statin doses, concomitant use of certain drugs (e.g. strong CYP3A4 inhibitors, amiodarone), Chinese ethnicity,^10^ and *SLCO1B1* genotype^11^ ^,^ ^12^—can increase the risk.^5^ ^,^ ^9^ ^,^ ^13^ As myopathy is rare, there is little reliable information about the independent relevance of these or other risk factors, or of the relative strength of their associations.^6^ ^,^ ^8^ ^,^ ^9^ ^,^ ^12^ ^,^ ^14^ ^,^ ^15^

This observational study aims to assess the relevance of independent risk factors for myopathy based on 171 systematically diagnosed cases among 58 390 simvastatin-treated participants from three large trial populations,^16–18^ and to examine the association between risk factors for simvastatin-related myopathy and risk of other muscle symptoms.

## Methods

### Study populations

The study population included 58 390 participants who received simvastatin: 9808 UK patients in the Heart Protection Study (HPS) trial (recruited 1994–97) allocated 40 mg simvastatin daily for ∼5 years (ISRCTN48489393)^16^; 11 538 UK patients in the SEARCH trial (recruited 1998–2001) allocated simvastatin 20 or 80 mg daily for ∼7 years (ISRCTN74348595)^17^; and 25 673 European and Chinese patients in the HPS2-THRIVE trial (recruited 2007–10) given simvastatin 40 mg daily (and randomly allocated niacin-laropiprant or matching placebo) for ∼4 years, along with an additional 11 371 patients who were not randomized but who received simvastatin 40 mg daily plus niacin-laropiprant during a 7–10 week pre-randomization ‘run-in’ period (ISRCTN29503772).^18^ Other pre-randomization periods in these trials (during which a further seven myopathy cases occurred) were not included due to a lack of comparable risk factor data.

Scheduled follow-up visits were conducted at least 6-monthly after initiation of study simvastatin and additional visits conducted when patients reported muscle symptoms. At each visit, participants were asked a directed question about any new unexplained muscle pain or weakness and alanine transaminase (ALT) was measured. Creatine kinase was measured: if muscle symptoms were reported in HPS; at each follow-up visit irrespective of symptoms in SEARCH; and if muscle symptoms were reported or routinely measured ALT was >1.5× ULN in HPS2-THRIVE.

Approval was obtained from the ethics committees of the participating institutions for each study, and all participants gave written informed consent.^16–18^

### Risk factors

Information was collected on baseline demographics and other patient characteristics. Exposure to concomitant medications, allowing for changes in medications over time, including stopping and restarting, was estimated. Medication start and stop times were taken as the mid-points between the times at which medication was reported to have been not taken and reported to have been taken. Genetic data were available in a subset of 9239 participants across all three studies.

### Myopathy and other muscle pain or weakness

Myopathy was prospectively defined as otherwise unexplained muscle pain or weakness with CK >10× ULN (within 28 days). The majority of myopathy cases presented to their managing clinician between scheduled clinic visits, and all cases were adjudicated by a clinician at the study co-ordinating centre. A report during follow-up of new unexplained muscle pain or weakness that was not associated with a diagnosis of myopathy (within 28 days) was defined as ‘other muscle symptoms’. A subset of these individuals with CK >5 ≤ 10× ULN was also examined.

### Statistical methods

Associations between potential risk factors and myopathy were estimated using Cox proportional hazards models. Participants were censored at the earliest of: reported non-compliance with study simvastatin (based on study drug dispensing records or patient reports), use of amiodarone (as simvastatin dose restrictions were imposed when used in combination with amiodarone in SEARCH and HPS2-THRIVE), myopathy, death, or study end. Stepwise selection (threshold *P* < 0.01) was used to identify a model including all independent risk factors and estimate their joint regression (i.e. independent) coefficients. Internal cross-validation was subsequently undertaken, using 171 non-overlapping groups comprising a single myopathy case and ∼1/171 of the additional participants selected randomly with proportional representation for study, simvastatin dose and ethnicity.^19^ For individuals in each of the 171 groups, joint regression coefficients for the independent risk factors were estimated using data from the other 170 independent groups. A combined score representing the effects of all the independent risk factors was then calculated for each participant, based on the sum of the independent risk factors weighted by the cross-validation derived joint regression coefficient. Associations with myopathy were estimated across thirds of this combined score. Among the 9239 genotyped individuals, the associations of *SLCO1B1* rs4149056 genotype with myopathy were estimated using logistic regression models adjusted for ethnicity and simvastatin dose. The expected numbers of myopathy cases per 10 000 person-years were estimated based on the underlying hazard at 6 months to represent the first year of study simvastatin, and at 2 years to represent longer-term treatment. Additional details of the calculation of the combined risk score and absolute risk are provided in the Supplementary material online, *Supplementary Methods*.

Confidence intervals based on floated variances are presented in figures for variables with more than two levels in order to allow direct comparisons between different groups (avoiding restriction to a single arbitrary reference group).^20^ Estimates and standard confidence intervals are given for direct two-way comparisons. Analyses were performed using SAS (v9.3).

## Results

### Baseline characteristics

Study participants for the HPS, SEARCH, and HPS2-THRIVE trials were pre-selected to be at high risk of cardiovascular events, with most individuals having a history of myocardial infarction, ischaemic stroke, or peripheral vascular disease (Supplementary material online, *Table S1*). Use of statins before study entry varied considerably reflecting periods of recruitment and regional differences; from none in HPS (1994–97) to 48% of Chinese and 96% of European participants in HPS2-THRIVE (2006–10). The simvastatin regimen provided in each study was at least equivalent in LDL-lowering efficacy to that received prior to study entry.

**Table 1:** Rates of myopathy by study, treatment, ethnicity, and time

		Time on study statin (overall mean 3.4 years)							
		≤1 year		>1 year		Overall	
Study and treatment	Ethnicity	Events/ person-years	Rate (SE) per 10 000 person-years	Events/ person-years	Rate (SE) per 10 000 person-years	Events/ person-years	Rate (SE) per 10 000 person-years
HPS							
Simvastatin 40 mg	European	3/9399	3 (2)	5/33 840	1 (1)	8/43 239	2 (1)
SEARCH							
Simvastatin 80 mg	European	21/5423	39 (8)	22/26 458	8 (2)	43/31 881	13 (2)
Simvastatin 20 mg	European	1/5461	2 (2)	1/25 236	0 (0)	2/30 697	1 (0)
HPS2-THRIVE							
Simvastatin 40 mg	Chinese	60/11 303	53 (7)	49/31 074	16 (2)	109/42 377	26 (2)
Simvastatin 40 mg	European	4/14 950	3 (1)	5/33 377	1 (1)	9/48 327	2 (1)
All participants		89/46 536	19 (2)	82/149 985	5 (1)	171/196 521	9 (1)

### Myopathy and other muscle symptoms

During 196 521 person-years of exposure to study simvastatin across the three studies, representing a mean 3.4 years of treatment, 171 participants developed myopathy, including 14 cases in whom there was evidence of more marked muscle damage (i.e. CK > 40× ULN) as well as end-organ damage (defined prospectively as rhabdomyolysis). Of the 131 individuals who had myopathy and at least one scheduled follow-up visit within the previous 28 days (when they would have been explicitly asked about any muscle symptoms), 96 (73%) had not reported muscle pain or weakness prior to the diagnosis of myopathy. The mean time from initiation of study simvastatin to myopathy was 18 months, with 36% of cases occurring in the first 6 months of treatment. The rate of myopathy per 10 000 person-years was 9 overall, but it was higher in the first year of treatment vs. later years (19 vs. 5), in Chinese vs. European individuals (26 vs. 2 with simvastatin 40 mg daily), and in those receiving higher doses (13 vs. 1 with simvastatin 80 mg vs. 20 mg daily doses; *Table 1*).

Creatine kinase was measured at every study visit in the SEARCH study, and less extreme CK elevations (CK > 5 ≤ 10× ULN) than required for the definition of myopathy were detected on 7 (0.2%) of 4495 visits at which muscle symptoms were reported and 102 (0.1%) of 171 090 visits at which they were not. In contrast to myopathy, reports of muscle symptoms other than myopathy (i.e. pain or weakness but without CK elevations >10× ULN) were extremely common, occurring at least once during follow-up in 26% (15 208/58 390) of participants, with an overall rate of 981 events per 10 000 person-years (Supplementary material online, *Table S2*).

**Table 2:** *SLCO1B1* and risk of myopathy among 9239 genotyped participants

		rs4149056 genotypes in myopathy cases/controls			C-allele carrier frequency in controls (%)	Odds ratio (95% CI) (C-allele carriers vs. non-carriers)	*P*-value
Study and treatment	Ethnicity	TT	CT	CC			
HPS							
Simvastatin 40 mg	European	4/6149	3/2190	1/191	28	2.58 (0.61–10.93)	0.18
SEARCH							
Simvastatin 80 mg	European	12/102	13/27	9/4	23	6.03 (2.73–13.94)	1.4 × 10^−5^
HPS2-THRIVE							
Simvastatin 40 mg	Chinese	53/352	27/89	8/5	21	2.47 (1.52–4.00)	2.4 × 10^−4^
All participants^a^							
Simvastatin 40 mg or 80 mg	Any	69/6603	43/2306	18/200	28	3.10 (2.09–4.59)	1.5 × 10^−8^

Odds ratios for myopathy for C-allele carriers vs. non-carriers are presented. This compares individuals with either CT or CC genotypes to individuals with TT genotype. Among all participants, odds ratio per C allele: 2.94, 95% CI: 2.15–4.03, *P* = 1.4 × 10^−11^.

a Adjusted for ethnicity and statin dose.

### Independent risk factors for simvastatin-related myopathy

**Figure 1:**
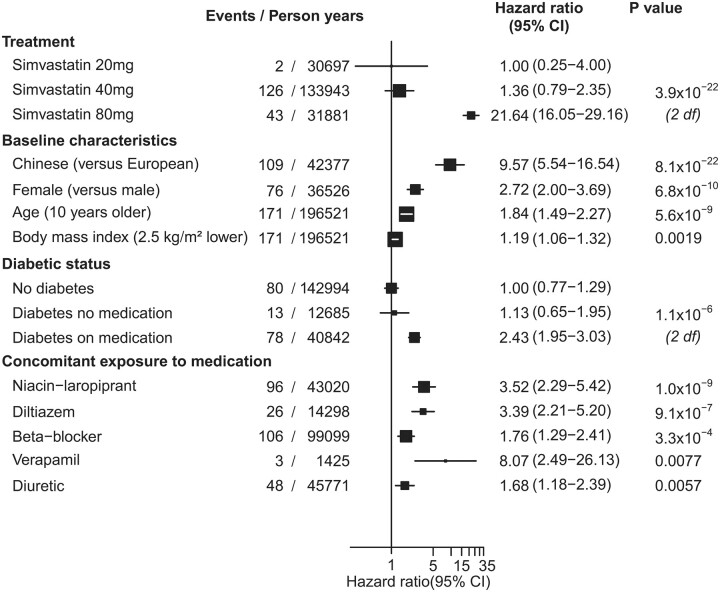
Associations of independent risk factors with myopathy, among 58 390 simvastatin-treated HPS, SEARCH, and HPS2-THRIVE participants. Figure shows independent effect estimates from a joint regression model (with floating absolute risks for variables with >2 groups). Box sizes are weighted by the number of events. *P*-values are 1 degree of freedom (df) tests unless otherwise stated.

Of the independent risk factors identified for myopathy (*Figure 1*), simvastatin dose (with doses other than 40 mg daily only used by European individuals, and 20 and 80 mg doses only used in the SEARCH trial) was the strongest predictor, with >20-fold higher risk among those receiving simvastatin 80 mg vs. 20 mg daily after allowance for other risk factors. In contrast, there was no significant difference in risk between patients who received 40 or 20 mg doses [hazard ratio (HR): 1.36, 95% CI: 0.31–6.05, *P* = 0.68). Chinese participants (who all received 40 mg simvastatin daily) had a ∼10-fold risk of myopathy compared to European participants, and older age, lower body mass index, and being female were each independently associated with higher risks. In addition, independent of the other risk factors identified, diabetic individuals receiving hypoglycaemic medication were at over twice the risk of myopathy compared with non-diabetic individuals (HR, 2.43; 95% CI: 1.73–3.41), whereas diabetic individuals not receiving any such medication were at comparable risk to those without diabetes (HR, 1.13; 95% CI: 0.62–2.06). Concomitant use of certain other medications also independently influenced myopathy risk: verapamil was associated with an eight-fold higher risk; niacin-laropiprant (mostly driven by events in Chinese individuals) and diltiazem with more than three-fold higher risks; and beta-blockers and diuretics with about 65–75% higher risks.

These independent risk factors were combined to form a weighted myopathy risk score reflecting the characteristics of each participant (see Supplementary material online, *Supplementary Methods*). Supplementary material online, *Table S3* shows the effects of each of the possible risk factors that were considered (after adjustment for simvastatin dose and ethnicity), as well as the effects after adjustment for the combined risk score, in order to demonstrate that no additional variables added materially to the risk of myopathy. For example, glomerular filtration rate estimated by the MDRD equation (eGFR) was significantly associated with myopathy in analyses adjusted for simvastatin dose and ethnicity (*P* = 5.6 × 10^−7^) but was not independent of the risk score (*P* = 0.08).

### Distinction between myopathy and other muscle symptoms

**Figure 2:**
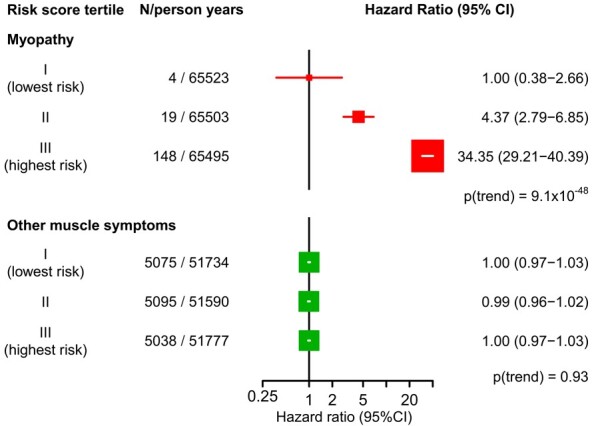
Risk of myopathy and other muscle symptoms, by myopathy risk score tertiles. Hazard ratios with 95% confidence intervals (CIs) based on cross-validated estimates are presented with floating absolute risks.

The combined myopathy risk score had a median of 7.2 (IQR 6.1–8.0) in myopathy cases and 4.2 (IQR 3.1–5.6) in other participants. It was a very strong predictor of myopathy, with a 34-fold difference in myopathy risk between the top and bottom thirds (HR, 34.35; 95% CI: 12.73–92.69; *P* for trend across thirds = 9.1 × 10^−48^; *Figure 2*). Patients with muscle symptoms and less extreme CK elevations (CK > 5 ≤ 10× ULN; *n* = 62) than required for the definition of myopathy had only a 3.5-fold difference in risk between top and bottom thirds of the myopathy risk score (HR, 3.51; 95% CI: 1.74–7.09; *P* for trend across thirds = 6·1 × 10^−5^).

In contrast, among the large number of patients (*n* = 15 208) reporting any muscle symptoms while taking a statin other than those confirmed to be myopathy, there was no association with the myopathy risk score (HR, 1.00; 95% CI: 0.96–1.04; *P* for trend across thirds = 0.93; *Figure 2*). The associations between each of the independent variables and these other muscle symptoms are presented in Supplementary material online, *Table S4*.

### Genetic variation in *SLCO1B1*, and risk of myopathy and other muscle symptoms

The rs4149056 *SLCO1B1* functional variant previously associated with myopathy in the SEARCH study^12^ was examined in 130 myopathy cases vs. 9109 controls genotyped in the HPS, SEARCH, and THRIVE studies. Overall, individuals carrying an rs4149056 C allele were at three-fold higher risk of myopathy (odds ratio, 3.10; 95% CI: 2.09–4.59, *P* = 1.5 × 10^−8^; *Table 2*). The association was consistent in Chinese and European participants (*P* for heterogeneity = 0.75) and independent of the combination of non-genetic risk factors identified. In contrast to the effect on myopathy, rs4149056 was not associated with other muscle symptoms (3035 cases vs. 6074 controls; odds ratio for C-allele carriers vs. non-carriers, 0.97; 95% CI: 0.89–1.06, *P* = 0.46; Supplementary material online, *Table S5*).

### Risk of myopathy with simvastatin 40 mg daily

The potential impact of common risk factor profiles on the absolute risk of myopathy due to simvastatin in our study populations is illustrated in Supplementary material online, *Figures S1 and S2*. Based on a standard 40 mg daily dose of simvastatin (not used in combination with diltiazem, niacin-laropiprant, or verapamil, which are all contraindicated in current practice), there was a seven-fold difference in myopathy risk between top and bottom thirds of the combined score (HR, 7.05; 95% CI: 1.61–30.82; *P* for trend across thirds = 3.2 × 10^−4^). In these individuals, the absolute risk of myopathy in European individuals ranged between <1 and 41 per 10 000 person-years across a range of common risk factor profiles during the first year; reducing by ∼40% during subsequent years of treatment (Supplementary material online, *Figure S1*). In Chinese individuals, the absolute risks were ∼10-fold higher than in European individuals (Supplementary material online, *Figure S2*).

## Discussion

The findings of this study have implications both to help guide safety monitoring with statin therapy, and to help ensure that patients do not stop their statin therapy due to mistakenly attributing common muscle symptoms to it. The absolute risk of myopathy due to standard statin regimens is low, but individuals can be identified who are at elevated risk by combining a number of independent risk factors in a myopathy risk score. The lack of association of this score with the much more common reports of other muscle symptoms is consistent with the randomized placebo-controlled evidence that statin therapy does not typically cause such symptoms (Take Home Figure). Consequently, CK should be measured in patients who report muscle symptoms on a statin and monitored in the small minority who are found to have moderately elevated CK levels, but otherwise they should be encouraged to continue taking their statin therapy.

**Take home figure:**
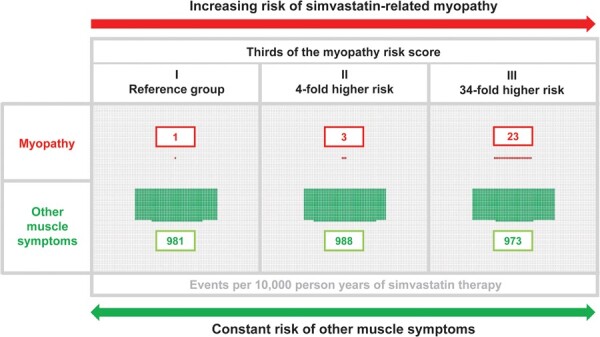
Risk of simvastatin-related myopathy does not predict risk of other muscle symptoms.

A number of hypotheses have been proposed as to why statins cause myopathy (including changes in mitochondrial function, energy production, and muscle protein degradation), but the mechanisms remain unclear.^21^ ^,^ ^22^ The risk of myopathy appears to depend on the levels of a statin in the circulation, as indicated by its association with genetic variation in *SLCO1B1*, which encodes OATP1B1 and can reduce the transport of statins from the circulation into the liver, leading to decreased statin clearance and higher blood levels.^10^ ^,^ ^12^ ^,^ ^23^ In addition to the dose of different statins, Chinese ethnicity, niacin, and verapamil are all recognized to increase blood statin levels.^10^ The higher risk of myopathy associated with lower body mass index (highly correlated with lower body surface area) in the present study may reflect the impact of lower blood volume or slower drug clearance in those with smaller body size, leading to higher blood levels. Poor renal function increases blood statin levels, but there was no independent effect of eGFR on myopathy risk in our analyses; this may reflect the limited range of eGFR represented in the present study (an average of about 90 mL/min/1.73 m^2^ and with fewer than 3% of participants having eGFR < 45 mL/min/1.73 m^2^). It is unclear whether diabetes itself (with the lack of an association when hypoglycaemic drugs were not being used reflecting less severe diabetes) or particular hypoglycaemic medications (e.g. some may affect OATP transportation, in which case the association reflects a drug-interaction) affect risk of myopathy.^24^ Furthermore, additional risk factors not measured in this study may also affect blood statin levels and be relevant to myopathy risk (e.g. CYP3A4 inhibitors, thyroid disease, grapefruit juice).^14^

Myopathy has been reported with all statins and, as in the present analyses with simvastatin, the rate appears to be dose-dependent and related to statin blood levels.^25^ ^,^ ^26^ For example, in regulatory databases, higher doses of atorvastatin and rosuvastatin are associated with higher rates of myopathy.^25^ ^,^ ^27^ ^,^ ^28^ Indeed, cerivastatin was withdrawn from use because the myopathy rate during post-marketing surveillance with approved doses was much higher (especially when combined with gemfibrozil) than with other statins.^7^ Shared mechanisms of statin uptake into the liver, metabolism, and pharmacology (e.g. with regards to hepatic processing and OATP transportation) suggest that, although different statins confer different absolute risks of myopathy, factors that increase the relative risk of myopathy would likely be shared. For example, variation in *SLCO1B1* has been associated with varying degrees of higher statin blood levels in pharmacokinetic studies with different statins: 221% increase in simvastatin (40 mg) exposure, 144% in atorvastatin (20 mg) exposure, 117% in rosuvastatin (40 mg) exposure, and 70% in rosuvastatin (10 mg) exposure for CC vs. TT genotypes.^23^ Such studies also indicate that people of East Asian ancestry exhibit higher blood levels for rosuvastatin, atorvastatin, and simvastatin acid, suggesting a class effect.^29^ Owing to a lack of comparable data with similar large numbers of consistently defined cases of myopathy, the relevance of the independent risk factors assessed in the present study has not been evaluated for any other statin. However, it would seem plausible that risk factors for simvastatin-related myopathy would increase the relative risk of myopathy with other statins in relation to their effect on statin blood levels.

Reports of muscle pain or weakness with statin therapy are very common (a quarter of the participants in the present study reported them on at least one occasion) and are often cited as a reason for discontinuing treatment. Patients’ perceptions of the possibility of muscle-related problems with statins may influence their likelihood of reporting them (i.e. the ‘nocebo’ effect).^5^ ^,^ ^7^ For example, in ASCOT-LLA, the rates of reported muscle symptoms did not differ between participants on atorvastatin 10 mg daily or on matching placebo while blinded to their randomized assignment, whereas during the subsequent ‘open-label’ phase, those who were receiving much the same statin therapy were 40% more likely to report such symptoms than those who did not.^30^ The lack of association in the present study of muscle pain or weakness in the absence of marked CK elevations with a risk score associated with more than 30-fold differences in the relative risk of myopathy is consistent with the lack of an excess of such symptoms with statin therapy in the randomized blinded comparisons.^5^ ^,^ ^7^ This finding supports the conclusion that these commonly reported muscle symptoms are not part of a continuum with simvastatin-related myopathy, but instead represent a nocebo effect.

The results of this study are relevant to the treatment of millions of patients worldwide. Simvastatin continues to be widely used: for example, it is the second most widely prescribed statin in the US with over 56 million prescriptions in 2017^4^; and, in the UK, nearly 22 million simvastatin prescriptions were dispensed in 2019.^3^ The absolute risk of statin-related myopathy on standard statin regimens is typically low; 2 per 10 000 patients per annum with simvastatin 40 mg daily in the present study. However, the absolute risk is influenced considerably by patient characteristics and by concomitant treatment with certain commonly used medications. For example, in this study population, certain risk factor combinations confer differences in risk comparable to doubling simvastatin dose from 40 to 80 mg daily (i.e. ~20-fold higher than average). It should be noted, however, that the benefits of statin treatment typically far outweigh any statin-related myopathy risks even in people who are at the highest risk of myopathy.^1^

A better understanding of factors affecting the risk of myopathy due to statin therapy could help guide safer prescribing in people at higher risk of it (e.g. use of lower doses of more potent statins, perhaps in combination with other LDL-lowering agents) and support more regular monitoring strategies for those in higher-risk groups. When patients do report muscle-related symptoms, measurement of CK levels (particularly during the first year of therapy or after an increase in dose or the addition of interacting medications) is warranted not only to detect the rare cases of myopathy but also to identify individuals with moderately elevated levels who may be more likely to develop myopathy. In addition, the much larger number of patients who do not have elevated CK levels can be reassured that their symptoms are not likely to be a pharmacologic consequence of their statin treatment; therefore, they should not stop their statin therapy and put themselves at increased risk of a heart attack or a stroke.

## Supplementary Material

ehaa574_Supplementary_Appendix
