# Supplementary material for: Independent risk factors for simvastatin-related myopathy and relevance to different types of muscle symptom
Source: Eur Heart J. 2020 Jul 23;41(35):3336–42. doi: 10.1093/eurheartj/ehaa574 (PMC7544537; doi:10.1093/eurheartj/ehaa574)
Supplement: ehaa574_Supplementary_Appendix [file ehaa574_supplementary_appendix.pdf]

Independent risk factors for simvastatin-related myopathy and relevance to different types of muscle symptom

J C Hopewell et al.

SUPPLEMENTARY APPENDIX

- **SUPPLEMENTARY METHODS**
- **SUPPLEMENTARY FIGURES**

Figure S1: Estimated absolute risk of myopathy per 10,000 person years in individuals of European ethnicity receiving simvastatin 40mg daily, by duration of study statin.

Figure S2: Estimated absolute risk of myopathy per 10,000 person years in individuals of Chinese ethnicity receiving simvastatin 40mg daily, by duration of study statin.

- **SUPPLEMENTARY TABLES**

Table S1: Treatment and baseline characteristics among 58,390 simvastatin-treated HPS, SEARCH and HPS2-THRIVE participants, by study.

Table S2: Rates of muscle pain or weakness by study, treatment, ethnicity and time.

Table S3: Associations of baseline characteristics with risk of myopathy among 58,390 simvastatin-treated HPS, SEARCH and HPS2-THRIVE participants.

Table S4: Associations of baseline characteristics with risk of muscle pain or weakness among 58,390 simvastatin-treated HPS, SEARCH and HPS2-THRIVE participants.

Table S5: *SLCO1B1* and risk of muscle pain or weakness among 9109 genotyped participants.

SUPPLEMENTARY METHODS

- **Data availability statement**

Proposals for data access will be considered in accordance with the trial protocols. The procedures for accessing the data are available at https://www.ndph.ox.ac.uk/data-access.

- **Estimation of absolute risk of myopathy and the combined risk score**

The absolute risk of myopathy per 10,000 person-years based on the populations in the present study was estimated as follows:

Events per 10,000 person years (shorter term treatment)

$= (1- 0.999984 ^\wedge (MRS_i /1.62646)) \times 10{,}000 / 0.5$

Events per 10,000 person years (longer term treatment)

$= (1- 0.999962 ^\wedge (MRS_i /1.62646)) \times 10{,}000 / 2.0$

Where the individual participant myopathy risk score (MRS_i) for the risk factor combinations shown in Figures S1 and S2, based on simvastatin 40mg daily (with no concomitant use of contraindicated medications including verapamil, diltiazem, and niacin-laropiprant), can be calculated as follows:

MRS_i = 1.934699 (constant)

+ 0.0610358 × patients age (in years)

- 0.0680302 × patients body mass index (kg/m^2)

+ 0.999787 if patient is female

+ 0.889089 if patient is diabetic on medication

+ 0.120865 if patient is diabetic but on no medication

+ 0.565850 if patient is on beta-blockers

+ 0.516883 if patient is on diuretics

+ 2.25887 if patient is of Chinese ethnicity

Figure S1: Estimated absolute risk of myopathy per 10,000 person years in individuals of European ethnicity receiving simvastatin 40mg daily, by duration of study statin.

a) First year of study simvastatin 40mg daily

	Male						Age	Female					
	Non-diabetic			Diabetic treated with medication				Non-diabetic			Diabetic treated with medication		
Body mass index (kg/m²)	30	25	20	30	25	20		30	25	20	30	25	20
**Concomitant medications**													
None*	<1	<1	<1	<1	1	2	55	<1	1	2	2	3	4
Beta-blocker	<1	<1	1	1	2	3		2	2	3	4	5	7
Diuretic	<1	<1	1	1	2	3		1	2	3	4	5	7
Beta-blocker + diuretic	<1	1	2	2	3	5		3	4	5	6	9	12
None*	<1	<1	1	1	2	3	65	2	2	3	4	5	8
Beta-blocker	1	1	2	3	4	5		3	4	6	7	10	13
Diuretic	<1	1	2	2	3	5		3	4	5	7	9	13
Beta-blocker + diuretic	2	2	3	4	6	8		5	7	9	11	16	23
None*	1	2	2	3	4	5	75	3	4	6	7	10	14
Beta-blocker	2	3	4	5	7	9		5	7	10	13	18	25
Diuretic	2	3	4	4	6	9		5	7	10	12	17	24
Beta-blocker + diuretic	3	5	6	8	11	15		9	12	17	21	30	41

b) Longer-term study simvastatin 40mg daily

	Male						Age	Female					
	Non-diabetic			Diabetic treated with medication				Non-diabetic			Diabetic treated with medication		
Body mass index (kg/m²)	30	25	20	30	25	20		30	25	20	30	25	20
**Concomitant medications**													
None*	<1	<1	<1	<1	<1	<1	55	<1	<1	1	1	2	3
Beta-blocker	<1	<1	<1	<1	1	2		<1	1	2	2	3	4
Diuretic	<1	<1	<1	<1	1	2		<1	1	2	2	3	4
Beta-blocker + diuretic	<1	<1	1	1	2	3		2	2	3	4	5	7
None*	<1	<1	<1	<1	1	2	65	<1	1	2	2	3	5
Beta-blocker	<1	<1	1	2	2	3		2	2	3	4	6	8
Diuretic	<1	<1	1	1	2	3		2	2	3	4	6	8
Beta-blocker + diuretic	1	2	2	3	4	5		3	4	6	7	10	14
None*	<1	<1	1	2	2	3	75	2	3	4	4	6	8
Beta-blocker	1	2	2	3	4	6		3	4	6	8	11	15
Diuretic	1	2	2	3	4	5		3	4	6	7	10	14
Beta-blocker + diuretic	2	3	4	5	7	9		5	7	10	13	18	25

Estimated annual risk of event colour coding: Green (<0.1%), amber (0.1% to 1%), red (≥1%). Estimates in the first year of study statin are based on the absolute risk at 6 months, and estimates for longer term study statin are based on the absolute risk at 2 years.

*None is defined as no concomitant use of risk factor medications (i.e. beta-blockers, diuretics, diltiazem, niacin-laropiprant, or verapamil)

Figure S2: Estimated absolute risk of myopathy per 10,000 person years in individuals of Chinese ethnicity receiving simvastatin 40mg daily, by duration of study statin.

a) First year of study simvastatin 40mg daily

	Male							Female					
	Non-diabetic			Diabetic treated with medication			Age	Non-diabetic			Diabetic treated with medication		
Body mass index (kg/m²)	30	25	20	30	25	20		30	25	20	30	25	20
**Concomitant medications**													
None*	3	4	6	7	10	15	55	8	12	16	20	28	40
Beta-blocker	5	8	11	13	18	26	55	15	20	29	35	50	70
Diuretic	5	7	10	12	17	25	55	14	19	27	34	47	66
Beta-blocker + diuretic	9	13	18	22	31	43	55	24	34	48	59	83	117
None*	6	8	11	14	19	27	65	15	21	30	37	52	73
Beta-blocker	10	14	19	24	34	47	65	27	38	53	65	91	128
Diuretic	9	13	19	23	32	45	65	26	36	50	62	87	122
Beta-blocker + diuretic	17	23	33	40	56	79	65	45	63	89	109	153	215
None*	10	15	20	25	35	49	75	28	39	55	68	96	134
Beta-blocker	18	26	36	44	62	87	75	49	69	97	120	168	235
Diuretic	17	24	34	42	59	83	75	47	66	93	114	160	224
Beta-blocker + diuretic	30	43	60	74	104	146	75	83	116	163	200	281	393

b) Longer-term study simvastatin 40mg daily

	Male							Female					
	Non-diabetic			Diabetic treated with medication			Age	Non-diabetic			Diabetic treated with medication		
Body mass index (kg/m²)	30	25	20	30	25	20		30	25	20	30	25	20
**Concomitant medications**													
None*	2	3	4	4	6	9	55	5	7	10	12	17	24
Beta-blocker	3	5	6	8	11	15	55	9	12	17	21	30	42
Diuretic	3	4	6	8	11	15	55	8	12	16	20	28	40
Beta-blocker + diuretic	5	8	11	13	18	26	55	15	21	29	36	50	70
None*	3	5	7	8	12	16	65	9	13	18	22	31	44
Beta-blocker	6	8	12	14	20	28	65	16	23	32	39	55	77
Diuretic	6	8	11	14	19	27	65	15	22	30	37	52	73
Beta-blocker + diuretic	10	14	20	24	34	48	65	27	38	53	65	92	128
None*	6	9	12	15	21	30	75	17	24	33	41	57	80
Beta-blocker	11	15	22	27	37	52	75	30	42	58	72	100	141
Diuretic	10	15	21	25	35	50	75	28	40	56	68	96	134
Beta-blocker + diuretic	18	26	36	44	62	87	75	50	69	97	120	167	233

Estimated annual risk of event colour coding: Green (<0.1%), amber (0.1% to 1%), red (≥1%). Estimates in the first year of study statin are based on the absolute risk at 6 months, and estimates for longer term study statin are based on the absolute risk at 2 years.

*None is defined as no concomitant use of risk factor medications (i.e. beta-blockers, diuretics, diltiazem, niacin-laropiprant, or verapamil)

Table S1: Treatment and baseline characteristics among 58,390 simvastatin-treated HPS, SEARCH and HPS2-THRIVE participants, by study.

	HPS	SEARCH	HPS2-THRIVE	
	European	European	Chinese	European
Study statin (daily)	simvastatin 40mg	simvastatin 20mg /simvastatin 80mg	simvastatin 40mg	simvastatin 40mg
Statin treated participants*	9808	11538	14755	22289
Person years exposed to study statin†	43239	62578	42377	48327
Additional lipid modification			+/- ezetimibe	+/- ezetimibe
**Baseline characteristics [mean (SD) or n(%)]**				
Age	64.1 (8.4)	64.2 (8.9)	63.2 (7.7)	66.0 (7.4)
Age range (10%-90%)	39-81 (51-74)	32-81 (52-75)	50-81 (53-74)	50-81 (56-76)
Female	2436 (25%)	1981 (17%)	3574 (24%)	3974 (18%)
Body mass index (kg/m²)	27.6 (4.4)	28.1 (4.1)	26.2 (3.2)	28.8 (5.0)
Alcohol drinker	5952 (61%)	7169 (62%)	1793 (12%)	13478 (60%)
Current or ex-smoker	7415 (76%)	8965 (78%)	8734 (59%)	15399 (69%)
Prior statin use	0 (0%)	8362 (72%)	7130 (48%)	21414 (96%)
Systolic blood pressure (mmHg)	145 (24)	137 (21)	143 (22)	144 (20)
Diastolic blood pressure (mmHg)	81.5 (12.2)	79.3 (11.7)	79.4 (12.2)	80.4 (10.9)
**Prior disease at entry [n(%)]**				
Myocardial infarction	4057 (41%)	5644 (49%)	9492 (64%)	15416 (69%)
Other coronary heart disease	2353 (24%)	4788 (41%)	1972 (13%)	2283 (10%)
Peripheral arterial disease	3261 (33%)	266 (2%)	726 (5%)	4400 (20%)
Ischemic stroke‡	1005 (10%)	346 (3%)	5569 (38%)	2421 (11%)
Diabetes	2750 (28%)	1170 (10%)	5742 (39%)	5375 (24%)
Treated hypertension	4000 (41%)	4824 (42%)	9356 (63%)	13759 (62%)
**Medication at entry [n(%)]**				
Calcium channel blockers				
Verapamil	160 (2%)	135 (1%)	0 (0%)	0 (0%)
Diltiazem	1050 (11%)	1269 (11%)	561 (4%)	973 (4%)
Amlodipine	577 (6%)	1227 (11%)	1021 (7%)	3155 (14%)
Other	1317 (13%)	523 (5%)	3361 (23%)	1575 (7%)
Diuretic	2351 (24%)	2683 (23%)	1732 (12%)	6279 (28%)
ACEi or ARB	1863 (19%)	4742 (41%)	6331 (43%)	15408 (69%)
Beta-blockers	2572 (26%)	5645 (49%)	7583 (51%)	14134 (63%)
Alpha-blockers	151 (2%)	365 (3%)	86 (1%)	1717 (8%)
Nitrates	3057 (31%)	5122 (44%)	5880 (40%)	5515 (25%)
Aspirin	6271 (64%)	10577 (92%)	12565 (85%)	19027 (85%)
Warfarin	446 (5%)	510 (4%)	81 (1%)	1827 (8%)
Bronchodilator	722 (7%)	869 (8%)	36 (0%)	1714 (8%)
Traditional Chinese medicine			5820 (39%)	373 (2%)
**Biochemical measurements [mean (SD)]**				
Total cholesterol (mg/dl)	155 (32)	164 (28)	121 (21)	133 (22)
LDL-cholesterol (mg/dl)	76.6 (25.0)	96.8 (23.5)	58.0 (15.8)	66.8 (17.1)
Apolipoprotein B (g/L)	77.4 (19.0)	90.0 (16.7)	64.1 (13.5)	69.8 (13.9)
HDL-cholesterol (mg/dl)	40.5 (12.2)	40.1 (13.7)	41.3 (9.3)	46.2 (12.4)
Apolipoprotein A1 (g/L)	122 (20)	135 (22)	139 (21)	151 (25)
Triglycerides (mg/dl)	147 (96)	171 (107)	122 (71)	129 (78)
Creatinine (mg/dl)§	1.06 (0.23)	1.01 (0.22)	0.84 (0.22)	0.92 (0.24)
log10(urine albumin:creatinine ratio)§			0.12 (0.60)	0.00 (0.55)
eGFR (ml/min/1.73m²)§	73.4 (16.8)	79.7 (18.2)	98.12 (29.5)	89.9 (33.3)
Creatine kinase (IU/L)§	113 (73)	130 (79)	90 (51)	110 (70)
Alanine transaminase (IU/L)§	21.6 (8.9)	24.9 (9.6)	20.6 (9.1)	23.3 (9.5)

ACEi or ARB: ACE inhibitors or angiotensin receptor blockers; eGFR: Estimated glomular filtration rate

* Includes the pre-randomization run-in period of HPS2-THRIVE during which participants received study statin (+/- ezetimibe) + niacin-laropiprant.

† Censoring at myopathy.

‡ For SEARCH and HPS this is stroke not known to be haemorrhagic.

§ Measurements made on entry into the study.

Table S2: Rates of muscle pain or weakness by study, treatment, ethnicity and time.

		Time on study statin (overall mean 3.4 years)					
		≤1 year		>1 year		Any	
**Study and treatment**	**Ethnicity**	**Events / person years**	**Rate (SE) per 10,000 person years**	**Events / person years**	**Rate (SE) per 10,000 person years**	**Events / person years**	**Rate (SE) per 10,000 person years**
**HPS**							
Simvastatin 40mg	European	1440 / 8765	1643 (43)	1816 / 24917	729 (17)	3256 / 33681	967 (17)
**SEARCH**							
Simvastatin 80mg	European	944 / 4957	1905 (62)	1429 / 17789	803 (21)	2373 / 22746	1043 (21)
Simvastatin 20mg	European	919 / 4989	1842 (61)	1309 / 17446	750 (21)	2228 / 22435	993 (21)
**HPS2-THRIVE**							
Simvastatin 40mg	Chinese	1190 / 10764	1106 (32)	1927 / 24497	787 (18)	3117 / 35261	884 (16)
Simvastatin 40mg	European	1877 / 14254	1317 (30)	2357 / 26723	882 (18)	4234 / 40977	1033 (16)
**All participants**		6370 / 43728	1457 (18)	8838 / 111372	780 (8)	15208 / 155100	981 (8)

Individuals with myopathy are excluded from these analyses

Table S3: Associations of baseline characteristics with risk of myopathy among 58,390 simvastatin-treated HPS, SEARCH and HPS2-THRIVE participants.

			Adjusted for treatment and ethnicity†		Adjusted for the myopathy risk score‡	
	Events (%)	Person years (%)	Hazard ratio (95% CI)	P value§	Hazard ratio (95% CI)	P value§
**Treatment and ethnicity***						
Simvastatin 20mg	2 (1%)	30697 (16%)	1.00 (0.25-4.00)	1.4×10^{-10}		
Simvastatin 40mg	126 (74%)	133943 (68%)	11.25 (9.27-13.65)	(2 df)		
Simvastatin 80mg	43 (25%)	31881 (16%)	21.16 (15.68-28.56)			
Chinese vs European	109 (64%)	42377 (22%)	5.97 (4.33-8.23)	2.4×10^{-29}		
**Baseline characteristics**						
Age (65+ versus <65)	111 (65%)	94923 (48%)	2.30 (1.68-3.16)	9.2×10^{-8}		
Age* (10 years older)	171	196521	2.06 (1.68-2.53)	7.1×10^{-13}		
Female* (versus male)	76 (44%)	36526 (19%)	3.27 (2.42-4.42)	2.3×10^{-13}		
Body mass index* (2.5 kg/m^2 lower)	171	196521	1.14 (1.02-1.27)	0.021		
Alcohol drinker (versus non-drinker)	43 (25%)	101035 (51%)	0.69 (0.46-1.02)	0.059	1.04 (0.72-1.51)	0.84
Current or ex-smoker (versus never)	87 (51%)	139899 (71%)	0.51 (0.38-0.69)	1.7×10^{-5}	0.87 (0.64-1.19)	0.38
Prior statin use (versus none)	101 (59%)	112494 (57%)	1.17 (0.85-1.59)	0.33	0.94 (0.70-1.28)	0.72
Systolic blood pressure (10 mmHg higher)	171	196458	1.05 (0.98-1.12)	0.19	1.01 (0.95-1.08)	0.75
Diastolic blood pressure (5 mmHg higher)	171	196458	0.87 (0.82-0.93)	3.1×10^{-5}	0.96 (0.90-1.02)	0.19
**Prior disease**						
Myocardial infarction	96 (56%)	110089 (56%)	0.88 (0.65-1.20)	0.42	0.98 (0.72-1.33)	0.90
Other coronary heart disease	44 (26%)	46144 (23%)	1.38 (0.96-1.98)	0.086	1.00 (0.70-1.41)	0.98
Peripheral arterial disease	16 (9%)	25275 (13%)	1.75 (1.02-3.00)	0.058	1.47 (0.87-2.46)	0.17
Ischemic stroke	52 (30%)	28249 (14%)	1.25 (0.88-1.79)	0.22	1.36 (0.98-1.90)	0.07
Treated hypertension	110 (64%)	99388 (51%)	1.41 (1.03-1.94)	0.032	1.05 (0.76-1.44)	0.77
**Diabetic status***						
Diabetic not treated with medication	13 (8%)	12685 (6%)	1.17 (0.64-2.14)			
Diabetic treated with medication	78 (46%)	40842 (21%)	2.75 (1.98-3.83)	1.2×10^{-8}		
**Concomitant exposure to other medications**						
Verapamil*	3 (2%)	1425 (1%)	5.00 (1.57-15.95)	0.029		
Diltiazem*	26 (15%)	14298 (7%)	3.18 (2.08-4.87)	2.8×10^{-6}		
Amlodipine	23 (13%)	21288 (11%)	1.67 (1.08-2.60)	0.032	1.54 (0.99-2.39)	0.067
Other calcium channel blockers	29 (17%)	21493 (11%)	1.04 (0.69-1.56)	0.86	1.03 (0.69-1.54)	0.89
Niacin-laropiprant						
Europe	5 (8%)	22738 (15%)	0.90 (0.31-2.60)	0.84		
China	91 (83%)	20282 (48%)	4.40 (2.62-7.38)	3.0×10^{-10}		
All*	96 (56%)	43020 (22%)	3.22 (2.10-4.93)	1.1×10^{-8}		
Diuretic*	48 (28%)	45771 (23%)	2.15 (1.52-3.04)	3.7×10^{-5}		
ACEi or ARB	59 (35%)	65162 (33%)	1.12 (0.80-1.58)	0.50	0.95 (0.69-1.31)	0.77
Beta-blockers*	106 (62%)	99099 (50%)	1.55 (1.14-2.11)	0.0049	1.02 (0.75-1.40)	0.89
Alpha-blockers	5 (3%)	9417 (5%)	1.31 (0.53-3.23)	0.57	1.11 (0.45-2.71)	0.82
Nitrates	86 (50%)	68863 (35%)	1.70 (1.25-2.30)	6.4×10^{-4}	1.21 (0.89-1.63)	0.23
Aspirin	143 (84%)	162689 (83%)	0.86 (0.57-1.29)	0.48	0.85 (0.56-1.27)	0.43
Warfarin	5 (3%)	10397 (5%)	1.23 (0.50-3.03)	0.67	0.94 (0.39-2.30)	0.89
Bronchodilator	5 (3%)	12729 (6%)	0.93 (0.37-2.32)	0.88	0.68 (0.28-1.67)	0.37
Ezetimibe	36 (21%)	41506 (21%)	1.05 (0.72-1.55)	0.79	0.87 (0.60-1.27)	0.47
Traditional Chinese medicine	48 (28%)	16287 (8%)	1.36 (0.94-1.99)	0.11	1.26 (0.89-1.77)	0.20
**Biochemical measurements (units)**						
Total cholesterol‖ (20 mg/dl higher)	171	195945	0.91 (0.80-1.04)	0.18	0.95 (0.85-1.06)	0.33
LDL-cholesterol‖ (10 mg/dl higher)	171	195936	0.87 (0.80-0.95)	0.0022	0.95 (0.89-1.02)	0.17
Apolipoprotein B‖ (10 g/L higher)	169	193416	0.85 (0.76-0.95)	0.0042	0.93 (0.85-1.02)	0.13
HDL-cholesterol‖ (10 mg/dl higher)	171	195908	1.13 (1.00-1.29)	0.064	1.00 (0.88-1.13)	0.96
Apolipoprotein A_1‖ (10 g/L higher)	169	193443	1.05 (0.99-1.13)	0.12	0.98 (0.92-1.05)	0.56
Triglycerides‖ (20 mg/dl higher)	171	195950	0.99 (0.95-1.03)	0.70	0.99 (0.95-1.03)	0.68
Creatinine¶ (0.05 mg/dl higher)	165	195112	1.03 (0.99-1.06)	0.16	1.02 (0.99-1.05)	0.12
$\log_{10}$(urine albumin:creatinine ratio)¶	81	69518	1.54 (1.12-2.12)	0.012	1.22 (0.86-1.73)	0.28
eGFR¶ (10 ml/min/1.73m^2 lower)	165	195112	1.19 (1.11-1.28)	5.6×10^{-7}	1.06 (0.99-1.13)	0.08
Creatine kinase¶ (10 IU/L higher)	171	196521	0.96 (0.91-1.02)	0.14	1.01 (0.96-1.06)	0.65
Alanine transaminase¶ (5 IU/L higher)	171	196521	0.90 (0.82-0.99)	0.018	1.00 (0.92-1.09)	0.97

ACEi or ARB: ACE inhibitors or angiotensin receptor blockers, eGFR: Estimated glomular filtration rate
*Variables included in the myopathy risk score. † Except for treatment and ethnicity which are unadjusted. ‡ Adjusted for the myopathy risk score (comprising treatment and ethnicity, age, sex, body mass index, diabetic status, and exposure to verapamil, diltiazem, niacin-laropiprant, diuretics and beta-blockers). § P-value based on 1df unless otherwise stated. ‖ Measurements made on study statin (simvastatin 40mg in HPS and HPS2-THRIVE, simvastatin 20mg in SEARCH). ¶ Measurements made on entry into the study.

Table S4: Associations of baseline characteristics with risk of muscle pain or weakness among 58,390 simvastatin-treated HPS, SEARCH and HPS2-THRIVE participants.

			Adjusted for treatment and ethnicity[†]		Adjusted for the myopathy risk score[‡]	
	Events (%)	Person years (%)	Hazard ratio (95% CI)	P value[§]	Hazard ratio (95% CI)	P value[§]
**Treatment and ethnicity***						
Simvastatin 20mg	2 228 (15%)	22435 (14%)	1.00 (0.96-1.04)	1.3×10^{-11}	1.00 (0.95-1.05)	4.8×10^{-9}
Simvastatin 40mg	10607 (70%)	109919 (71%)	0.88 (0.86-0.91)		0.85 (0.83-0.87)	
Simvastatin 80mg	2373 (16%)	22746 (15%)	1.06 (1.02-1.10)		0.92 (0.95-1.05)	
Chinese vs European	3117 (20%)	35261 (23%)	0.84 (0.81-0.88)	6.6×10^{-18}	0.77 (0.73-0.81)	2.8×10^{-28}
**Baseline characteristics**						
Age (65+ versus <65)	7546 (50%)	75273 (49%)	1.03 (0.99-1.06)	0.10	1.03 (1.00-1.07)	0.046
Age* (10 years older)	15208	155100	1.02 (1.00-1.04)	0.072	1.02 (1.00-1.05)	0.021
Female* (versus male)	3187 (21%)	27881 (18%)	1.21 (1.16-1.26)	3.9×10^{-21}	1.21 (1.16-1.26)	4.9×10^{-20}
Body mass index* (2.5 kg/m^2 lower)	15208	155100	0.97 (0.96-0.98)	5.5×10^{-13}	0.96 (0.95-0.97)	3.1×10^{-19}
Alcohol drinker (versus non-drinker)	7938 (52%)	78809 (51%)	1.01 (0.98-1.05)	0.45	1.09 (1.05-1.12)	1.7×10^{-6}
Current or ex-smoker (versus never)	10796 (71%)	110018 (71%)	0.99 (0.95-1.02)	0.48	1.01 (0.98-1.05)	0.51
Prior statin use (versus none)	8987 (59%)	89159 (57%)	1.00 (0.97-1.03)	0.97	1.03 (1.00-1.07)	0.039
Systolic blood pressure (10 mmHg higher)	15205	155042	0.99 (0.98-1.00)	0.0016	0.98 (0.98-0.99)	2.2×10^{-5}
Diastolic blood pressure (5 mmHg higher)	15205	155042	0.98 (0.97-0.99)	3.4×10^{-8}	0.98 (0.97-0.99)	1.5×10^{-8}
**Prior disease**						
Myocardial infarction	8623 (57%)	87838 (57%)	1.00 (0.97-1.03)	0.88	0.98 (0.95-1.01)	0.24
Other coronary heart disease	3788 (25%)	34098 (22%)	1.18 (1.13-1.22)	4.2×10^{-16}	1.22 (1.18-1.27)	5.3×10^{-26}
Peripheral arterial disease	2274 (15%)	19767 (13%)	1.20 (1.15-1.26)	5.2×10^{-14}	1.17 (1.12-1.23)	8.1×10^{-12}
Ischemic stroke	1997 (13%)	23314 (15%)	0.90 (0.85-0.95)	3.2×10^{-5}	0.83 (0.79-0.87)	5.3×10^{-15}
Treated hypertension	7982 (52%)	78698 (51%)	1.07 (1.04-1.11)	1.2×10^{-5}	1.04 (1.01-1.08)	0.01
**Diabetic status***						
Diabetic not treated with medication	1042 (7%)	10306 (7%)	1.03 (0.97-1.10)		0.97 (0.91-1.03)	
Diabetic treated with medication	3155 (21%)	32410 (21%)	1.05 (1.01-1.09)	0.062	0.99 (0.95-1.03)	0.59
**Concomitant exposure to other medications**						
Verapamil*	118 (1%)	1051 (1%)	1.14 (0.95-1.36)	0.17	1.19 (0.99-1.43)	0.068
Diltiazem*	1248 (8%)	10604 (7%)	1.20 (1.13-1.27)	1.7×10^{-9}	1.24 (1.17-1.32)	1.9×10^{-12}
Amlodipine	1674 (11%)	16382 (11%)	1.05 (1.00-1.11)	0.043	1.07 (1.01-1.12)	0.013
Other calcium channel blockers	1627 (11%)	17259 (11%)	1.00 (0.95-1.06)	0.94	0.95 (0.90-1.00)	0.045
Niacin-laropiprant						
Europe	2034 (17%)	19840 (17%)	0.97 (0.92-1.02)	0.26	0.89 (0.85-0.94)	8.2×10^{-6}
China	1460 (47%)	17204 (49%)	1.01 (0.94-1.08)	0.83	0.86 (0.79-0.93)	3.8×10^{-4}
All*	3494 (23%)	37044 (24%)	0.97 (0.93-1.01)	0.13	0.87 (0.84-0.91)	7.8×10^{-11}
Diuretic*	3580 (24%)	35090 (23%)	1.06 (1.02-1.10)	0.0051	1.08 (1.04-1.12)	6.1×10^{-5}
ACEi or ARB	5328 (35%)	53049 (34%)	1.06 (1.02-1.10)	0.0025	0.99 (0.96-1.03)	0.76
Beta-blockers*	7714 (51%)	78049 (50%)	1.01 (0.98-1.04)	0.49	1.01 (0.98-1.04)	0.48
Alpha-blockers	729 (5%)	7048 (5%)	1.10 (1.02-1.19)	0.014	1.13 (1.05-1.22)	0.0017
Nitrates	5730 (38%)	52101 (34%)	1.19 (1.16-1.24)	2.5×10^{-25}	1.21 (1.17-1.25)	3.0×10^{-29}
Aspirin	12613 (83%)	128401 (83%)	0.99 (0.94-1.03)	0.5	1.00 (0.96-1.05)	0.85
Warfarin	779 (5%)	8085 (5%)	0.99 (0.92-1.07)	0.81	1.02 (0.94-1.09)	0.68
Bronchodilator	1037 (7%)	9390 (6%)	1.13 (1.06-1.20)	2.8×10^{-4}	1.17 (1.10-1.25)	1.2×10^{-6}
Ezetimibe	3510 (23%)	34912 (23%)	1.00 (0.96-1.04)	0.84	0.95 (0.92-0.99)	0.0086
Traditional Chinese medicine	1276 (8%)	13173 (8%)	1.13 (1.06-1.22)	3.2×10^{-4}	0.94 (0.89-1.00)	0.058
**Biochemical measurements (units)**						
Total cholesterol[‖] (20 mg/dl higher)	15155	154624	1.01 (1.00-1.02)	0.072	1.04 (1.03-1.05)	1.5×10^{-12}
LDL-cholesterol[‖] (10 mg/dl higher)	15156	154611	1.01 (1.00-1.01)	0.17	1.03 (1.02-1.03)	1.2×10^{-13}
Apolipoprotein B[‖] (10 g/L higher)	14933	152590	1.01 (1.00-1.02)	0.021	1.04 (1.03-1.04)	2.7×10^{-14}
HDL-cholesterol[‖] (10 mg/dl higher)	15149	154593	0.99 (0.97-1.00)	0.044	0.98 (0.97-1.00)	0.012
Apolipoprotein A_1[‖] (10 g/L higher)	14933	152619	1.00 (0.99-1.00)	0.22	0.99 (0.99-1.00)	0.062
Triglycerides[‖] (20 mg/dl higher)	15155	154629	1.01 (1.00-1.01)	2.5×10^{-5}	1.01 (1.01-1.01)	6.4×10^{-10}
Creatinine[¶] (0.05 mg/dl higher)	15073	153983	1.00 (1.00-1.00)	0.56	1.00 (1.00-1.01)	0.029
$\log_{10}$(urine albumin:creatinine ratio)[¶]	5410	58193	1.05 (1.00-1.10)	0.041	1.04 (0.99-1.09)	0.15
eGFR[¶] (10 ml/min/1.73m^2 lower)	15073	153983	1.02 (1.01-1.03)	6.5×10^{-4}	1.03 (1.02-1.03)	7.6×10^{-8}
Creatine kinase[¶] (10 IU/L higher)	15208	155100	1.01 (1.00-1.01)	0.016	1.01 (1.01-1.01)	3.9×10^{-6}
Alanine transaminase[¶] (5 IU/L higher)	15208	155100	1.00 (0.99-1.01)	0.68	1.01 (1.00-1.02)	0.025

ACEi or ARB: ACE inhibitors or angiotensin receptor blockers, eGFR: Estimated glomular filtration rate.
* Variables included in the myopathy risk score. † Except for treatment and ethnicity which are unadjusted. ‡ Adjusted for the myopathy risk score (comprising treatment and ethnicity, age, sex, body mass index, diabetic status, and exposure to verapamil, diltiazem, niacin-laropiprant, diuretics and beta-blockers). § P-value based on 1df unless otherwise stated. ‖ Measurements made on study statin (simvastatin 40mg in HPS and HPS2-THRIVE, simvastatin 20mg in SEARCH). ¶ Measurements made on entry into the study.
Muscle pain or weakness events (i.e. other muscle symptoms) exclude myopathy.

Table S5: *SLCO1B1* and risk of muscle pain or weakness among 9109 genotyped participants

Study and treatment	Ethnicity	rs4149056 genotypes in cases / controls			C-allele carrier frequency in controls (%)	Odds ratio (95% CI) C-allele carriers vs non-carriers	P value
		TT	CT	CC			
**HPS**							
Simvastatin 40mg	European	2083/4066	705/1485	66/125	28%	0.95 (0.87-1.04)	0.28
**SEARCH**							
Simvastatin 80mg	European	36/66	13/14	2/2	20%	1.55 (0.78-3.11)	0.21
**HPS2-THRIVE**							
Simvastatin 40mg	Chinese	101/251	27/62	2/3	21%	1.12 (0.71-1.76)	0.61
**All participants***							
Simvastatin 40mg or 80mg	Any	2220/4383	745/1561	70/130	28%	0.97 (0.89-1.06)	0.46

*Adjusted for ethnicity and statin dose. Individuals with myopathy are excluded from these analyses.

Odds ratios for C-allele carriers versus non-carriers are presented. This compares individuals with either CT or CC genotypes to individuals with TT genotype. Among all participants, odds ratio per C allele: 0.97, 95% CI: 0.88 to 1.05, P=0.43.
